# Total neoadjuvant treatment to increase the clinical complete response rate for distal locally advanced rectal cancer (TESS): A study protocol of a prospective, open‐label, multicenter, single‐arm, phase 2 trial

**DOI:** 10.1002/cam4.6034

**Published:** 2023-05-08

**Authors:** Shuang Liu, XiaoZhong Wang, YeZhong Zhuang, ShouMin Bai, XiaoJun Wu, YiJing Ye, HuiLong Luo, HaiNa Yu, QiaoXuan Wang, Hui Chang, ZhiFan Zeng, PeiQiang Cai, ZhiZhong Pan, YuanHong Gao, Gong Chen, WeiWei Xiao

**Affiliations:** ^1^ Department of Radiation Oncology Sun Yat‐sen University Cancer Center Guangzhou China; ^2^ Sun Yat‐sen University Cancer Center State Key Laboratory of Oncology in South China, Collaborative Innovation Center for Cancer Medicine Guangzhou China; ^3^ Department of General Surgery Shantou Central Hospital Shantou China; ^4^ Department of Abdominal Surgery Cancer Hospital of Shantou University Medical College Shantou China; ^5^ Department of Radiation Oncology Sun Yat‐sen Memorial Hospital, Sun Yat‐sen University Guangzhou China; ^6^ Department of Colorectal Surgery Sun Yat‐sen University Cancer Center Guangzhou China; ^7^ Department of Radiation Oncology Zhongshan People's Hospital Zhongshan China; ^8^ Department of Medical Imaging and Interventional Radiology Sun Yat‐sen University Cancer Center Guangzhou China

**Keywords:** clinical trials, neoadjuvant chemotherapy, rectal cancer, surgical oncology

## Abstract

**Background:**

Standard treatment of locally advanced rectal cancer (LARC) was neoadjuvant chemoradiotherapy (CRT), followed by total mesorectal excision (TME). Total neoadjuvant treatment (TNT), a new concept, attempts to deliver both systemic chemotherapy and neoadjuvant CRT prior to surgery. Patients treated with neoadjuvant chemotherapy were more likely to show higher tumor regression. The objective of this trial was to increase complete clinical rate (cCR) for LARC patients by optimizing tumor response, using TNT regimen as compared to conventional chemoradiotherapy. TESS, a prospective, open‐label, multicenter, single‐arm, phase 2 study, is underway.

**Methods:**

Main inclusion criteria include cT3‐4aNany or cT1‐4aN+ rectal adenocarcinoma aged 18‐70y; Eastern Cooperative Oncology Group (ECOG) performance 0–1; location ≤5 cm from anal verge. Ninety‐eight patients will receive 2 cycles of neoadjuvant chemotherapy Capeox (capecitabine + oxaliplatin) before, during, and after radiotherapy 50Gy/25 fractions, before TME (or other treatment decisions, such as Watch and Wait strategy) and adjuvant chemotherapy capecitabine 2 cycles. Primary endpoint is the cCR rate. Secondary endpoints include ratio of sphincter preservation strategy; pathological complete response rate and tumor regression grade distribution; local recurrence or metastasis; disease‐free survival; locoregional recurrence‐free survival; acute toxicity; surgical complications; long‐term anal function; late toxicity; adverse effect, ECOG standard score, and quality of life. Adverse events are graded per Common Terminology Criteria for Adverse Events V5.0. Acute toxicity will be monitored during antitumor treatment, and late toxicity will be monitored for 3 years from the end of the first course of antitumor treatment.

**Discussion:**

The TESS trial aims to explore a new TNT strategy, which is expected to increase the rate of cCR and sphincter preservation rate. This study will provide new options and evidence for a new sandwich TNT strategy in patients with distal LARC.

## 1 BACKGROUND

Neoadjuvant chemoradiotherapy (CRT) followed by total mesorectal excision (TME) with or without adjuvant chemotherapy is the standard treatment for locally advanced rectal cancer (LARC). The standard treatment has been reported to reduce local recurrence for LARC patients. However, there was no improvement in the distant metastasis.^1^ Additionally, TME surgery may lead to urinary and sexual function impairment due primarily to the removal of the rectum. Thus, perioperative treatment strategies are developed to decrease surgical morbidity and improve quality of life in surviving patients, such as the changing sequences of chemotherapy and radiotherapy and prolonging the interval between surgery and radiotherapy.^2^, ^3^

Total neoadjuvant treatment (TNT), a new concept, shifts all or part of adjuvant chemotherapy to the neoadjuvant phase. Two TNT paradigms have emerged: neoadjuvant induction chemotherapy followed by CRT (NeoCT‐CRT) and CRT followed by neoadjuvant consolidation chemotherapy (CRT‐NeoCT). Many trials assessing TNT have shown obvious advantages in increased treatment compliance along with improved tumor response.^4^, ^5^ Findings from a study of 628 patients treated at Memorial Sloan‐Kettering Cancer Center (MSKCC) demonstrated that patients in the TNT arm received greater percentages of the planned oxaliplatin and fluorouracil prescribed dose compared with chemoradiotherapy and planned adjuvant chemotherapy arm. And this trial also found improved tumor responses: 36% of patients in TNT arm had either pathologic complete response (pCR) or clinical complete response (cCR) compared with chemoradiotherapy and planned adjuvant chemotherapy arm (21%).^6^ RAPIDO and PRODIGE‐23 trials showed that TNT strategy could increase the pCR rate and reduce the risk of distant metastasis.^7^, ^8^, ^9^, ^10^ And for patients with a cCR after neoadjuvant CRT, National Comprehensive Cancer Network (NCCN) guidelines suggest “Watch and Wait (W&W)” strategy.^11^ It is widely acknowledged that “W&W” strategy offers the potential to avoid surgery‐associated morbidity and bowel, urinary, and sexual dysfunction that can permanently impair the patient's quality of life.

The lengthening of the interval between CRT and TME has been reported to achieve more pCR.^2^, ^12^ Recent trials showed that the surgical interval of 10 weeks would attain the best pCR rates without impacting survival outcomes.^13^ The addition of chemotherapy in the surgical interval may have greater tumor response, even achieving higher cCR rates and providing potential for the W&W strategy. So, the surgery can be avoided to reduce the risks of postoperative complications. However, the longer surgical interval in “CRT‐NeoCT” TNT model may aggravate pelvic fibrosis and edema making it more difficult to ensure the integrity of surgical resection of the mesorectum, thereby increasing the difficulty of TME surgery and reducing the quality of surgery.^14^, ^15^, ^16^, ^17^

The efficacy and safety of double‐drugs oxaliplatin‐based concurrent neoadjuvant chemoradiotherapy is still under debate.^18^, ^19^, ^20^, ^21^ However, based on our center's experience in clinical practice and several prospective clinical trials, it has been shown to significantly increase tumor response and to be well tolerated in LARC patients.^3^, ^22^, ^23^, ^24^ The FOWARC study also demonstrated that the combination of oxaliplatin and fluorouracil with long‐course radiotherapy was acceptably tolerated and resulted in a higher pCR rate compared with single‐agent fluorouracil plus radiotherapy.^25^

Thus, our study designs a new sandwich model of TNT, in which 2 cycles of Capeox are added before, during, and after radiotherapy, followed by TME (or other treatment decisions, such as W&W strategy) and adjuvant chemotherapy capecitabine 2 cycles. This sandwich TNT model with double‐drugs before, during, and after radiotherapy would be more compact. And we assess whether it increases the cCR rate and how to influence the proportion of various sphincter preservation strategies in patients with LARC compared with conventional neoadjuvant chemoradiotherapy.

## 2 METHODS/DESIGN

### 2.1 Study overview

**FIGURE 1:**
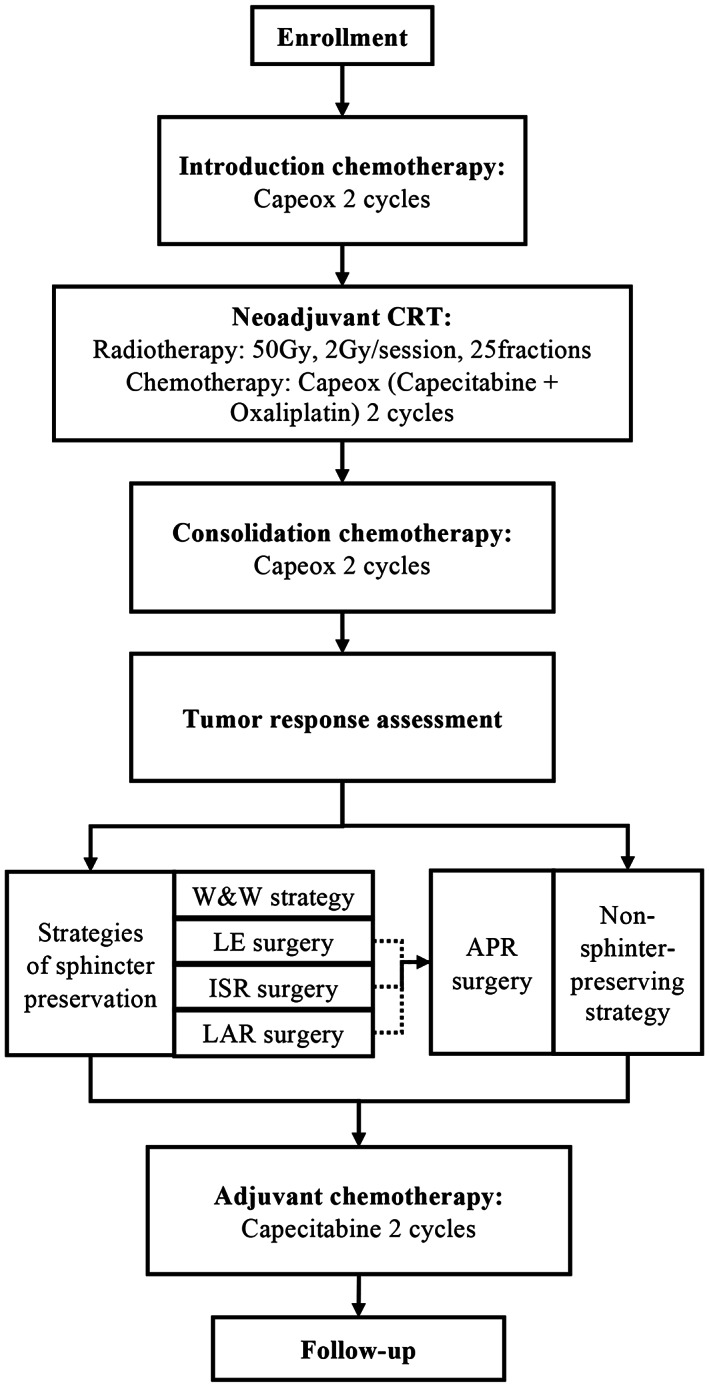
Study design of TESS trial. APR, abdominal‐perineal resection; CRT, chemoradiotherapy; ISR, intersphincteric resection; LAR, low anterior resection; LE, local excision; W&W strategy, watch and wait strategy.

This study is a prospective, multicenter, single‐arm, phase 2 clinical trial, comparing a new sandwich TNT regimen to conventional chemoradiotherapy in the cCR rate of patients with LARC. Our study aimed to increase the cCR rate for LARC patients who are unable or not sure to undergo sphincter‐preserving surgery at initial diagnosis. The study design is shown in Figure 1. The primary endpoint is the cCR rate. This study is conducted in full conformance with the principles of the Declaration of Helsinki.

### 2.2 Participants

#### 2.2.1 Screening of eligibility criteria

Eligible patients are screened by the investigators at each participating center. All patients are required to have a complete workup of LARC within 2 weeks before the treatments, including medical history review, the collection of personal data, currently taken medications and treatments, physical examination (height, weight, vital signs, anorectal digital examination, lymph node examination, and physical examination of the chest and abdomen), neurological examination, routine blood tests, urinal test, routine biochemistry, Eastern Cooperative Oncology Group (ECOG) performance score, biopsy pathology, CT and MRI of the chest/abdomen, pelvic MRI and endoscopic ultrasonography or transrectal color Doppler ultrasound‐guided electronic colonoscopy.

#### 2.2.2 Inclusion criteria

Patients eligible for inclusion in this study should meet all the following criteria:

1. Rectal adenocarcinoma
2. cT3‐4aNany or cT1‐4aN+
3. Distal location ≤5 cm from the anal verge
4. No distant metastasis
5. No gastrointestinal obstruction or relieved obstruction
6. No previous surgery of the rectum, no previous chemotherapy, no previous pelvic radiation, no previous biotherapy
7. ECOG 0–1
8. Expected survival length ≥2 years
9. Age 18–70
10. Sufficient bone marrow, renal and liver function:
  1. WBC >4000/mm^3^
  2. PLT >100,000/mm3
  3. Hb >10 g/dL
  4. Creatinine <1.5 N
  5. Bilirubinemia <1.5 mg/dL
  6. AST and ALT ≤1.5 N
11. Effective contraception during the study
12. Patient and doctor have signed informed consent
13. Sphincter‐preserving surgery is not applicable or not sure by colorectal surgeon's evaluation

#### 2.2.3 Exclusion criteria

Patients eligible for this study must not meet any of the following criteria:

1. cT4b
2. Lateral lymph node metastasis
3. Severe arrhythmia, cardiac dysfunction (NYHA grade III or IV)
4. Uncontrollable severe hypertension
5. a history of HIV, hepatitis B virus or hepatitis C virus infection
6. Active severe infection
7. Distant metastasis
8. Cachexia, organ dysfunction
9. Previous pelvic radiotherapy or chemotherapy
10. Multiple primary cancers
11. Epileptic seizures
12. Malignant history within 5 years, except cervical carcinoma in situ or cutaneous basal cell carcinoma
13. Persons deprived of liberty or under guardianship
14. Certain or suspicious allergy to research drug
15. Contraindications for chemotherapy and/or radiotherapy
16. Impossibility for compliance to follow‐up
17. Pregnant or breast‐feeding woman
18. Peripheral neuropathy >grade 1
19. Severe renal, hepatic insufficiency (serum creatinine <30 mL/min)
20. Chronic intestinal inflammation and/or bowel obstruction
21. Substance abuse, psychological, medical, or social conditions that could interfere with the patient's participation in the study or evaluation of the results

#### 2.2.4 Drop‐out

A patient can be dropped out in cases as follows:

1. A patient's refusal for any reasons.
2. Patient's poor treatment compliance and failure to receive medication as per study protocol.

### 2.3 Sample size

After the amendment, the primary endpoint was changed from the sphincter preservation rate to the cCR rate, and therefore, the corresponding sample size was changed. Based on the results of a retrospective study in our center, the cCR rate after CRT for LARC patients was known to be 17%. Thus, we assume that the new sandwich TNT model would show a 13% improvement in cCR rate compared with the history control (17%–30%). Based on the superiority design, one‐sided proportion test and log‐rank test with α‐error of 0.025 and a power of 80% are conducted for the primary endpoint: the cCR rate. Allowing for a drop‐out rate of 10%, a total of 98 patients will need to be enrolled, eventually.

### 2.4 Randomization process

This study uses a single‐arm design; thus, no randomization is performed.

### 2.5 Therapeutic schemes

#### 2.5.1 Neoadjuvant chemotherapy

Neoadjuvant chemotherapy consisted of 6 cycles of Capeox (Oxaliplatin (130 mg/m^2^) as a 3‐h IV infusion on day 1, followed by capecitabine (1000 mg/m^2^) twice daily for 14 days every 3 weeks). In the third and fourth cycles of chemotherapy, patients receive oxaliplatin (100 mg/m^2^) and capecitabine (1000 mg/m^2^) concurrently with radiotherapy.

#### 2.5.2 Radiotherapy

Patients receive radiotherapy in 25 fractions of 2.0 Gy up to 50.0 Gy. Intensity‐modulated radiation therapy is conducted for the primary tumor, regional lymph nodes, and clinical target volume. The dose to gross tumor volume (GTV) and involved lymph nodes (GTVnd) is 50 Gy in 25 fractions, and the dose to clinical target volume (CTV) is 45 Gy in 25 fractions.

#### 2.5.3 Surgery

For other patients who did not assessed as cCR, the surgical treatment modality, including local excision (LE), intersphincteric resection (ISR), and low anterior resection (LAR), abdominal‐perineal resection (APR) should be determined after multidisciplinary team discussion. APR and LAR will be undertaken following principles of TME and tumor‐free for patients who are appropriate for undergoing resection. Surgical resection is performed 2–4 weeks after the end of preoperative treatment. The rectal resection will be performed by experienced professors with respect to colorectal cancer.

#### 2.5.4 Adjuvant chemotherapy

The use of two courses of capecitabine is recommended 21 days after R0 resection. In patients with positive margins confirmed by pathological examination, postoperative adjuvant chemoradiotherapy should be proposed. Clinicians could make an individualized treatment based on pathological results.

#### 2.5.5 Nonoperative therapy

Patients with cCR will be observed under the “W&W" strategy. cCR was defined according to the guideline of Chinese Society of Clinical Oncology (CSCO) colorectal cancer 2018.V1, including (1) digital rectal examination: normal; (2) white and flat mucosal scars under the endoscope, accompanied by peripheral capillary telangiectasia, without signs of malignant ulcers or nodules; and (3) high‐resolution MRI in T2 shows completely dark, without moderate intensity signals and lymph nodes; in DW phase, no tumor signal for B800‐B1000 and/or, in ADC shows little or no signal, and intestinal wall linear signals in the tumor area.

### 2.6 Follow‐up

All patients will be followed up as per protocol until 1.5 years (for the primary endpoint, not for others). For patients undergoing surgery, follow‐up will perform every 3 months within the first 3 years and every 6 months after 3 years up to 5 years, and annually thereafter. Follow‐up examination mainly consisted of physical examination, digital rectal examination, tumor markers detection, and ultrasonography. Enhanced pelvic MRI and contrast‐enhanced chest–abdomen CT should perform at least once a year. PET‐CT should be conducted if necessary. The patients who received “W&W” strategy should be more intensive followed up. Endoscope and pelvic MRI should be carried out in each follow‐up. Transrectal color Doppler ultrasound will also be conducted if necessary.

### 2.7 Study endpoints

Through investigator meetings, the primary endpoint was changed from the sphincter preservation rate to the cCR rate. Sphincter‐preserving strategies for LARC patients include sphincter‐preserving surgeries and the “W&W” strategy. However, serious postoperative complications, such as leaks or obstruction, may occur. The “W&W” strategy may be more appropriate for LARC patients with cCR after TNT to avoid surgery. For this reason, a new primary endpoint was formulated: the cCR rate. The change to this new endpoint was approved by the Ethical Committee of Sun Yat‐sen University Cancer Center (December 27, 2020) and amended on clinicaltrial.gov accordingly (January 23, 2022).

Secondary endpoints are ratio of sphincter preservation strategy, pathologic response of specimens after surgery according to the Tumor Regression Grading (TRG) system, local recurrence or metastasis, locoregional recurrence‐free survival (LRFS), disease‐free survival (DFS), acute toxicity, surgical complications, long‐term anal function, late toxicity, adverse events, ECOG performance score, and quality of life. Diagnosis of recurrence or progression can be made only when the clinical and laboratory findings meet at least one of the following criteria objective radiological recurrence or progression on radiological imaging (ultrasound, CT scan, MRI scan, and PET‐CT scan as indicated by the clinical picture), positive cytology or biopsy (in case of ascites, anastomotic recurrence, doubt on radiological imaging). LRFS is defined as the time interval from the randomization to the date of local relapse. DFS is calculated from the date of randomization to the date of disease recurrence, the appearance of new colorectal cancer or death. Acute and chronic toxicity will be recorded in the case report form (CRF). The grade of any adverse event will be recorded by the International Common Terminology Criteria for Adverse Events (CTCAE), V5.0.^26^

### 2.8 Statistical analysis

Continuous measures will be compared using the t test. Chi‐square test will be utilized to compare categorical variables. Survival analysis will be estimated using the Kaplan–Meier (log‐rank) test. The Cox proportional hazards model will be employed to identify the prognostic factors. The significance level is *p* < 0.05. The analysis will be performed with SPSS statistical software or R. Counts and percentage will be used to describe enumeration data, and the quantitative data will be described using means and 95% confidence.

## 3 DISCUSSION

At present, most researches of TNT treatment focused on improving pCR and survival.^7^, ^8^, ^9^ Our meta‐analysis showed that TNT strategy could increase the rate of pCR (OR = 1.77, 95% CI: 1.28–2.45, *p* = 0.0005) and reduce the risk of distant metastasis (HR = 0.81, 95% CI: 0.68–0.95, *p =* 0.012).^10^ The three‐drug combination regimen also had well treatment adherence and tolerance, according to the PRODIGE 23 trial.^9^ Despite the exponential increase in pCR rates in some studies,^7^, ^8^, ^9^ there was no improvement in organ preservation rates, as the study design did not aim to improve anal retention or avoidance of surgery. Further treatment optimization should be attentively directed toward improving organ preservation and quality of life in LARC.

The results of Organ Preservation in Rectal Adenocarcinoma (OPRA) trial showed a higher sphincter preservation rate in both induction group (NeoCT‐CRT) and the consolidation group (CRT‐NeoCT; 43% vs. 58%, *p* = 0.007), compared with standard neoadjuvant chemoradiotherapy (historical controls). There was no significant difference in toxicity between the induction group and the consolidation group, and there was no significant difference in DFS (77% vs. 78%, *p* = 0.63) and DMFS (82% vs. 84%, *p* = 0.83).^27^ Another prospective, single‐arm study, with cCR as the primary endpoint, includes cT3 or any cT1 cN+ or cT2 cN+ rectal cancer patients, now in progress.^28^

Similarly, the CAO/ARO/AIO‐12 trial showed that NeoCT‐CRT strategy has a higher pCR rate compared with CRT‐NeoCT strategy (17% vs. 25%) and the long‐term evaluations have not been reported.^29^ Due to the limited data, there is no consensus on which treatment is better for “NeoCT‐CRT” or “CRT‐NeoCT.” This study is currently the first investigation of the new sandwich TNT model of NeoCT‐CRT‐NeoCT. This model with 2 cycles of Capeox before, during, and after radiotherapy was a complete TNT paradigm. The time from the completion of radiotherapy to the evaluation of 8–10 weeks is appropriate, which can avoid the serious fibrosis caused by the long operation interval.^2^, ^30^, ^31^ In addition, two courses of chemotherapy during this period will consolidate the therapeutic effect effectively.

In fact, the assession criterion for cCR is still under discussion. We evaluate cCR by digital rectal examination, endoscope examination, and rectal MRI examination included T2WI and DWI sequences, according to CSCO guidelines.^32^ Although ESMO guidelines recommend the negative multipoint biopsy as an assessment criterion for cCR,^33^ its ideal evaluated timing and value remains controversial.^34^, ^35^ Therefore, rectal biopsy is not mandatory for cCR in our study.

Previous studies showed that “W&W" strategy was safe and feasible for cCR patients, with similar survival results and better quality of life, compared with those who received TME surgery.^36^, ^37^, ^38^, ^39^, ^40^, ^41^ Our recent study also demonstrated that there was no significant difference on 3‐year DMFS (W&W group vs. surgical group = 88% vs. 89%, *p* = 0.874).^42^ Another closer analysis showed that low‐risk cCR patients (CA19‐9 <35 U/mL and CEA <5 ng/mL) would benefit from TME surgery, compared with W&W (5‐year DMFS: 95.9% vs. 84.3%; *p* = 0.028).^43^

Positive lateral lymph node is a high‐risk factor of local recurrence and lymph node metastasis in LARC, especially low rectal cancer. Lateral lymph node metastasis, which usually invades adjacent organs, was reported to be 15%–30%.^44^, ^45^, ^46^, ^47^, ^48^ The JCOG0212 trial showed that the local recurrence would be reduced by lateral lymph node dissection (LLND) compared with the TME alone (5‐year local recurrence‐free survival: 87.7% vs. 82.4%, HR = 1.37, 95% CI: 0.97–1.93), especially in the lateral pelvis (7.4% vs. 12.6%, *p* = 0.024).^49^, ^50^, ^51^ However, because of the complexity of LLND and the increased incidence of complications, there is still tremendous controversy about its usage.^52^, ^53^ Some western studies considered lateral lymph node involvement indicated the presence of distant metastasis and use stronger neoadjuvant chemoradiotherapy to control tumor progression.^54^, ^55^, ^56^ Currently, there is still very limited data for the role of TNT and “W&W" strategy in LARC with lateral lymph node metastasis.^57^, ^58^ The RAPIDO study suggests that patients with lateral lymph node metastasis may also benefit from TNT strategy (3‐year disease‐related treatment failure: 33% vs. 25%, HR = 1.13, 95% CI: 0.64–2.01, *p* = 0.14). Therefore, we excluded patients with lateral lymph node metastasis in our study.

The TESS trial is designed with the aim of improving cCR and the sphincter preservation rate without compromising survival in distal LARC patients. In the TESS trial, we are exploring a completely new sandwich TNT paradigm, “NeoCT‐CRT‐NeoCT.” Patients will receive two cycles of neoadjuvant chemotherapy Capeox before, during, and after long‐course radiotherapy, and two cycles of adjuvant chemotherapy of capecitabine. This is expected to be an effective and safe treatment with better quality of life. The exploration of TNT versus standard chemoradiation will provide a new treatment option and evidence for the distant LARC.

## AUTHOR CONTRIBUTIONS

**Shuang Liu:** Conceptualization (lead); data curation (lead); methodology (lead); resources (equal); software (equal); writing – original draft (lead); writing – review and editing (lead). **XiaoZhong Wang:** Conceptualization (lead); data curation (lead); methodology (equal); project administration (equal); resources (equal); validation (equal). **YeZhong Zhuang:** Conceptualization (lead); data curation (lead); investigation (equal); methodology (equal); resources (equal); writing – review and editing (equal). **ShouMin Bai:** Conceptualization (equal); data curation (lead); formal analysis (lead); resources (equal); software (equal); supervision (equal); validation (equal). **XiaoJun Wu:** Conceptualization (equal); data curation (equal); formal analysis (equal); resources (equal); software (equal); supervision (equal); validation (equal). **YiJing Ye:** Conceptualization (equal); data curation (equal); formal analysis (equal); funding acquisition (equal); resources (equal); validation (equal). **HuiLong Luo:** Conceptualization (equal); data curation (equal); software (equal). **HaiNa Yu:** Conceptualization (equal); data curation (equal); resources (equal). **QiaoXuan Wang:** Conceptualization (equal); data curation (equal); validation (equal). **Hui Chang:** Conceptualization (equal); data curation (equal); formal analysis (equal); resources (equal). **ZhiFan Zeng:** Conceptualization (lead); data curation (equal); formal analysis (lead); funding acquisition (equal); investigation (equal); resources (lead); software (equal); supervision (equal); validation (lead); writing – original draft (equal); writing – review and editing (equal). **PeiQiang Cai:** Formal analysis (equal); funding acquisition (equal); methodology (equal); resources (equal); supervision (equal); validation (equal). **ZhiZhong Pan:** Conceptualization (equal); data curation (equal); formal analysis (equal); resources (equal); software (equal). **YuanHong Gao:** Conceptualization (equal); data curation (equal); formal analysis (equal); funding acquisition (equal); resources (equal); supervision (equal); validation (equal). **Gong Chen:** Conceptualization (lead); data curation (lead); formal analysis (lead); funding acquisition (equal); resources (lead); software (equal); supervision (equal); validation (lead); writing – original draft (equal); writing – review and editing (equal). **WeiWei Xiao:** Conceptualization (lead); data curation (lead); investigation (lead); resources (lead); supervision (lead); validation (lead).

## FUNDING INFORMATION

This work was supported by the 5010 Clinical Research Foundation of Sun Yat‐sen University (Grant number: 5010‐2018‐04).

## CONFLICT OF INTEREST STATEMENT

The authors declare that they have no conflict of interest.

## ETHICS APPROVAL STATEMENT

This study protocol and amendments have been approved by the Ethics Committee of Sun Yat‐sen University Cancer Center (5010‐2018‐04‐01, 5010‐2018‐04‐03). All patients will provide their written informed consent before any study‐related assessment.

## CLINICAL TRIAL REGISTRATION NUMBER

This study was registered on clinicaltrials.gov with NCT03840239.

## Data Availability

Not applicable.
